# Robot-assisted thoracoscopic resection of a posterior mediastinal schwannoma adjacent to the intervertebral foramen using Arista™ AH: A case report

**DOI:** 10.1097/MD.0000000000047817

**Published:** 2026-02-20

**Authors:** Chihiro Tando, Tsuyoshi Uchida, Tomohiro Hayata, Koshi Mobara, Yu Tsukahara, Aya Sugimura, Yuichiro Onuki, Hirochika Matsubara

**Affiliations:** a Department of General Thoracic Surgery, Yamanashi University, Chuo, Yamanashi, Japan; b Department of General Thoracic Surgery, Yamanashi Kosei Hospital, Yamanashi, Japan.

**Keywords:** hemostasis, mediastinal tumor, microporous polysaccharide hemisphere, robot-assisted thoracic surgery

## Abstract

**Rationale:**

Posterior mediastinal schwannomas near the intervertebral foramen pose surgical challenges owing to their proximity to critical neurovascular structures.

**Patient concerns:**

A 19-year-old woman presented with an abnormal shadow on a chest radiograph obtained during a routine health checkup.

**Diagnoses:**

Magnetic resonance imaging revealed a 3.5-cm mass in the right paravertebral region at the T3–T4 level, suggestive of schwannoma.

**Interventions:**

The tumor was resected via 4-port robot-assisted thoracic surgery. Intraoperatively, the intercostal nerve was found to traverse the tumor and was divided. Arista AH was used to control oozing near the intervertebral foramen.

**Outcomes:**

The patient recovered uneventfully and was discharged on postoperative day 8. Pathological examination confirmed schwannoma. She has remained recurrence-free for 12 months.

**Lessons:**

Robot-assisted thoracic surgery enables safe resection of tumors adjacent to the intervertebral foramen. Arista™ AH may provide effective hemostasis in anatomically sensitive areas.

## 1. Introduction

Posterior mediastinal tumors are frequently neurogenic in origin, and schwannomas are among the most common histological types. Histopathological diagnosis is essential to guide management, and complete surgical resection remains the mainstay of treatment for these lesions.^[1]^ Although many of these tumors can be safely resected using minimally invasive techniques, lesions located near the intervertebral foramen pose specific challenges owing to their proximity to critical neurovascular structures such as the intercostal vessels, dura mater, and spinal nerve roots.

Robot-assisted thoracic surgery (RATS) is gaining acceptance as a viable alternative to conventional thoracoscopic approaches, offering superior instrument articulation, tremor elimination, and stable three-dimensional visualization. These features make RATS particularly suitable for precise dissection in anatomically complex areas, such as the posterior mediastinum.^[2]^ In recent years, robotic systems have been applied not only to malignant thoracic diseases but also to benign neurogenic tumors, reflecting broader trends in the adoption of advanced minimally invasive technologies.

Herein, we describe a young woman with a posterior mediastinal schwannoma located adjacent to the intervertebral foramen, which was successfully resected using a robot-assisted thoracoscopic approach. An absorbable hemostatic agent, Arista AH, was applied intraoperatively near the foramen to control the oozing. Here, we discuss the surgical strategy and rationale for using this agent in anatomically delicate regions.

## 2. Case presentation

**Figure 1.**
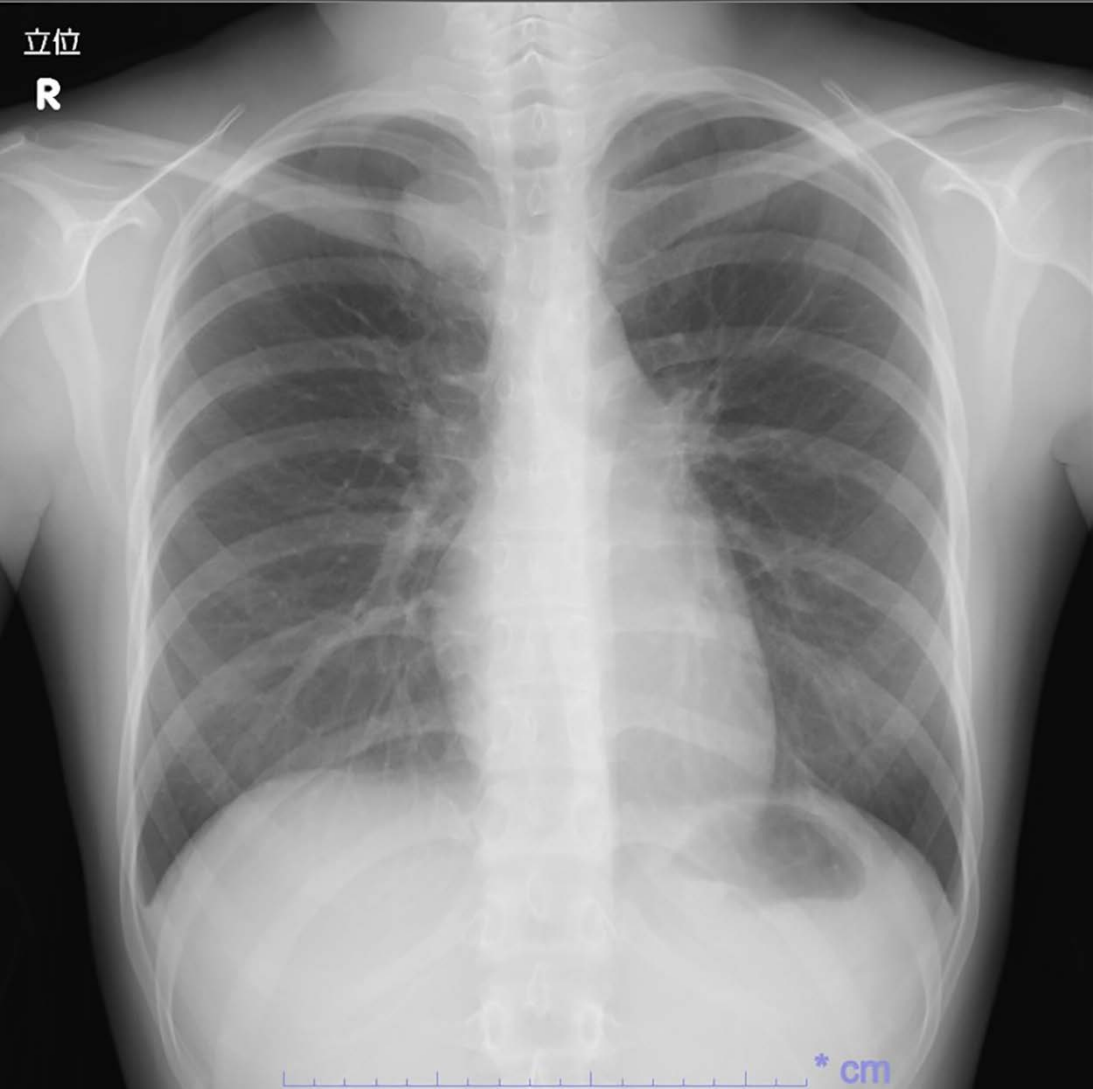
Chest radiograph. A well-defined mass of approximately 3 cm in size was observed on the mediastinal side of the right upper lung field. The silhouette sign was negative.

**Figure 2.**
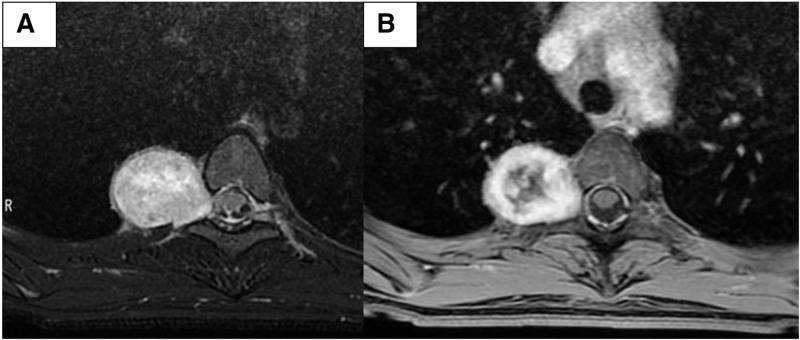
Preoperative magnetic resonance imaging (T2-weighted Image). (A) Pre-contrast-enhanced: a 3.5-cm hyperintense mass was noted at the T3–T4 level. (B) Post-contrast-enhanced: the mass demonstrates heterogeneous enhancement after contrast administration.

A 19-year-old woman was referred to our hospital for further evaluation of an abnormal opacity identified on chest radiography performed as part of a health checkup. Her medical history and physical examination results were unremarkable. Radiography revealed a well-circumscribed round mass in the right upper lung field (Fig. 1). Chest magnetic resonance imaging demonstrated a 3.5-cm nodule in the right paravertebral region at the T3–T4 level (Fig. 2A). The lesion was located adjacent to the intervertebral foramen, with associated scalloping of the adjacent rib, suggesting a slow-growing lesion. On T2-weighted imaging, the tumor appeared as a heterogeneous, hyperintense mass. Post-contrast imaging revealed internal heterogeneous enhancement, predominantly within areas of relatively low T2 signal, a characteristic finding of Antoni A areas observed in schwannomas (Fig. 2B).

**Figure 3.**
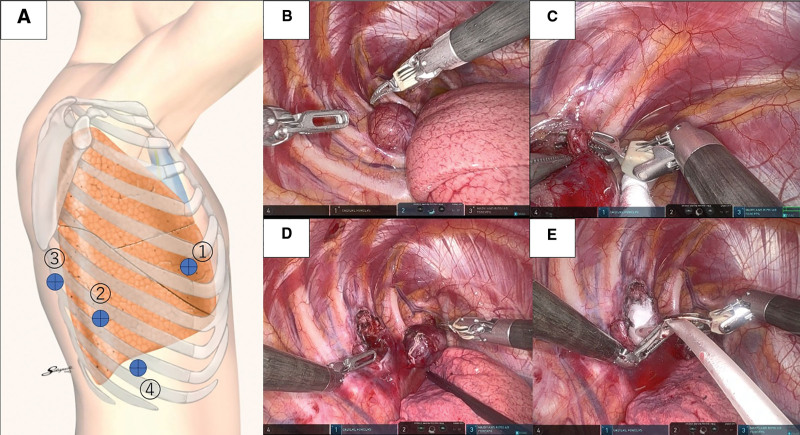
Intraoperative findings. (A) Port placement. Fifth intercostal space, anterior axillary line. Eighth intercostal space, posterior axillary line. Eighth intercostal space, below the inferior angle of the scapula. Nineth intercostal space, midaxillary line. (B) Intraoperative finding 1. There were no adhesions to the lung, and the tumor was encapsulated in a smooth membrane. (C) Intraoperative finding 2. The intercostal nerve traversed the tumor, making preservation difficult; therefore, the nerve was divided. (D) Intraoperative finding 3. The tumor was completely resected. No dural injury or cerebrospinal fluid leakage was observed; however, oozing was observed near this site. (E) Intraoperative finding 4. Hemostasis was achieved using Arista AH.

For diagnosis and treatment, the tumor was resected using a 4-port robot-assisted approach (Fig. 3A). The tumor was diagnosed as a schwannoma originating from the intercostal nerve, and enucleation was initially planned. However, during dissection, the third intercostal nerve was found to traverse the tumor, making preservation difficult. The nerve was clipped and divided (Fig. 3B–D). After the dissection, oozing was observed near the intervertebral foramen, and Arista AH was applied for hemostasis. The application was performed with the surgeon guiding the applicator while the assistant pushed the powder through it (Fig. 3E).

Although postoperative nausea persisted, the patient recovered uneventfully and was discharged on postoperative day 8. Pathological examination confirmed the diagnosis of schwannoma. No recurrence was observed during the 1-year postoperative follow-up period.

## 3. Discussion

This case highlights 2 key aspects. The first aspect is the usefulness of RATS in the resection of tumors adjacent to the intervertebral foramen. The tumor was in close proximity to the intervertebral foramen but could be safely resected en bloc via RATS. The resection of dumbbell-shaped tumors extending into the intervertebral foramen typically requires collaboration with the spinal surgeons. However, robot-assisted resection of these tumors has recently been reported.^[2]^ In our case, the tumor was adjacent to, but did not extend into, the foramen; allowing for complete resection via the thoracic approach alone.

Thoracoscopic surgery, although minimally invasive, may be limited by restricted visualization and instrument maneuverability, leading to less-precise dissection. In contrast, robot-assisted surgery offers advantages such as arm stabilization and tremor filtration, enabling meticulous and stable operations. Robot-assisted resection of posterior mediastinal tumors has previously been performed with preservation of critical structures such as the artery of Adamkiewicz, and this requires considerable skill in conventional thoracoscopic surgery.^[3]^ In the present case, surgery was also completed without injury to the surrounding vital structures, such as the intercostal vessels and dura mater.

The second aspect is the demonstration of the safe use of Arista AH for hemostasis close to the intervertebral foramen. Oozing was observed intraoperatively near the intervertebral foramen after tumor resection, and hemostasis was achieved by applying Arista AH without the need for additional intervention.

Arista AH consists of microporous polysaccharide hemispheres (MPHs) derived from purified potato starch and is used as an absorbable hemostatic agent. MPHs are a flowable powder that rapidly dehydrates the blood, thereby enhancing clot formation upon contact. MPHs absorb water and low-molecular-weight compounds, concentrate blood solids, and form scaffolds for fibrin clot formation. The resulting clot, which is composed of swollen MPH beads, platelets, and clotting factors, is reportedly more resilient than those formed naturally.^[4]^

Although oxidized regenerated cellulose is another absorbable hemostatic agent, its use near the intervertebral foramen is discouraged because of reported neurological complications such as lower extremity paralysis. The mechanism likely involves not only physical compression due to swelling from fluid absorption, but also acidification of the local environment and delayed absorption, which can take 4 to 8 weeks.^[5]^

In contrast, Arista AH is associated with minimal foreign-body reaction and is fully absorbed within 24 to 48 hours, making it safer to use near the intervertebral foramen.^[4,6]^ Based on these features, Arista AH may serve as a valuable adjunct in hemostasis during RATS. However, the direct application of Arista AH to neural structures is contraindicated, and its use should be avoided in cases where the membranous barrier of the foramen has been breached.

In summary, our findings suggest that RATS facilitates the safe resection of tumors adjacent to the intervertebral foramen and that Arista AH offers effective hemostasis with minimal foreign-body response, making it suitable for use in sensitive areas.

## Acknowledgments

We would like to thank Editage (www.editage.com) for the English language editing. The authors thank the patient who participated in the study.

## Author contributions

**Conceptualization:** Chihiro Tando, Tsuyoshi Uchida.

**Data curation:** Chihiro Tando, Tomohiro Hayata.

**Methodology:** Tsuyoshi Uchida, Yu Tsukahara.

**Resources:** Tsuyoshi Uchida, Koshi Mobara, Yuichiro Onuki.

**Supervision:** Hirochika Matsubara.

**Writing—original draft:** Chihiro Tando, Tsuyoshi Uchida.

**Writing – review & editing:** Tsuyoshi Uchida, Aya Sugimura.
